# Dexamethasone Modulates Global DNA Methylation and Oxidative DNA Damage in Glioblastoma Cell Lines

**DOI:** 10.3390/cells15191745

**Published:** 2026-09-24

**Authors:** Anna-Maria Barciszewska, Agnieszka Belter, Jakub F. Barciszewski, Patrick M. Perrigue, Kaja Jaskot, Małgorzata Giel-Pietraszuk, Mirosława Z. Naskręt-Barciszewska

**Affiliations:** 1 Intraoperative Imaging Unit, Department of Neurosurgery, Institute of Nervous System Diseases, Poznan University of Medical Sciences, Przybyszewskiego 49, 60-355 Poznań, Poland; ambarciszewska@ump.edu.pl; 2 Department of Neurosurgery, University Clinical Hospital, Przybyszewskiego 49, 60-355 Poznań, Poland; 3 Institute of Bioorganic Chemistry of the Polish Academy of Sciences, Noskowskiego 12, 61-704 Poznań, Poland; abelter@ibch.poznan.pl (A.B.);; 4 NanoBioMedical Center, Adam Mickiewicz University, Wszechnicy Piastowskiej 3, 61-614 Poznań, Poland

**Keywords:** dexamethasone, temozolomide, valproic acid, metformin, glioblastoma, 5-methylcytosine, 8-oxo-dG, oxidative DNA damage, epigenetics

## Abstract

Glioblastoma remains one of the most aggressive primary brain tumors, characterized by rapid progression, therapeutic resistance, and poor prognosis. Dexamethasone is routinely administered to patients with glioblastoma to reduce peritumoral edema and improve neurological symptoms. However, its broader molecular effects on tumor-cell biology and treatment response remain incompletely understood. This study examined the effects of dexamethasone alone and in combination with temozolomide, valproic acid, or metformin on glioblastoma cell viability, real-time proliferation, senescence-associated β-galactosidase activity, cell-cycle distribution, global DNA methylation, and oxidative DNA damage. Dexamethasone induced concentration- and time-dependent changes in global DNA methylation in glioma cells. In T98G cells, shorter exposure increased 5-methylcytosine, whereas longer incubation or selected combined treatment conditions reduced methylation, suggesting dynamic epigenetic remodeling. Low dexamethasone concentrations were associated with increased 5-methylcytosine content, also in temozolomide-containing conditions. In contrast, higher dexamethasone concentrations reduced global methylation. That effect was cell-line dependent. In SW1783 cells, high-dose dexamethasone caused a reduction in 5-methylcytosine amount as well as an increase in 8-oxo-2′-deoxyguanosine level. Dexamethasone also reduced real-time T98G cell proliferation after prolonged exposure, while temozolomide was the main compound associated with cell-cycle changes in T98G cells. Metformin displayed comparatively limited cytotoxicity under the tested conditions. Beyond dexamethasone’s well-established anti-edematous and anti-inflammatory role, it may also modify glioma redox and epigenetic states and influence cellular responses to temozolomide, valproic acid, and metformin. When considering these findings it is necessary to take into account a number of limitations. The upper limits of the DEX, TMZ, and MF concentration ranges were one to three orders of magnitude higher than the brain or CSF exposure levels that have been reported, and the experiments were carried out at standard culture oxygen concentrations (about 18–21% O_2_), which are much higher than the oxygen levels found in brain tissue (about 2–6% O_2_). Furthermore, the conclusions drawn from the T98G experiments only apply to this cell line and cannot be applied to glioblastoma without first being verified in other models.

## 1. Introduction

Understanding the cellular mechanisms of drug action is crucial for evaluating cancer transformation and for developing new therapeutic strategies. In recent years, increasing attention has focused on compounds that modulate epigenetic regulation and thereby contribute to controlling gene expression. This is particularly relevant in glioblastoma, where genetic alterations, epigenetic remodeling, oxidative stress, metabolic adaptation, and treatment resistance are closely interconnected.

DNA methylation of cytosine residues is one of the best-known epigenetic modifications in the mammalian genome. This change involves the covalent attachment of a methyl group from S-adenosylmethionine to the fifth carbon atom of cytosine, catalyzed by DNA methyltransferases, yielding 5-methylcytosine (m^5^C) [1,2]. DNA methylation helps regulate when, where, and for how long a gene is transcriptionally active or silenced [3]. In mammalian DNA, m^5^C occurs mainly in CpG dinucleotides and makes up about 1% of the human genome [4]. Although non-CpG methylation may also be present, it constitutes an important component of genome regulation [5,6,7].

The level of m^5^C in DNA reflects the cell’s developmental, differentiation, and metabolic status [4,8]. Because the carbon–carbon bond in m^5^C is highly stable, demethylation is complex. It may occur through enzymatic oxidation mediated by ten-eleven translocation proteins (TET) or through random oxidative processes associated with reactive oxygen species (ROS) [9,10,11,12]. Increased intracellular ROS may lead to DNA oxidation, mutations, genomic instability, altered proliferation, and chemotherapy resistance [13,14,15]. Thus, oxidative DNA damage and changes in DNA methylation may represent interconnected and dynamic processes of cancer-cell adaptation.

These processes are reflected by changes in at least two molecular markers: 5-methylcytosine, the main epigenetic marker of DNA, and 8-oxo-2′-deoxyguanosine (8-oxo-dG), a widely used marker of oxidative DNA damage. Cancer cells are often characterized by global DNA hypomethylation together with promoter-specific hypermethylation of tumor suppressor genes and other regulatory regions [16]. 8-oxo-dG is the product of the direct reaction of a guanine molecule with the hydroxyl radical (•OH). In addition to the binding of cytosine via Watson–Crick hydrogen bonds, 8-oxo-dG mispairs with adenine through Hoogsteen bonds and induces G–T transversions [17]. Oxidative stress appears to shift the cell’s redox balance, as ROS can contribute to oxidative DNA damage and may influence DNA methylation and demethylation dynamics [11,18]. Although global m^5^C analysis does not identify locus-specific methylation changes, it provides an integrated measure of the genome’s overall epigenetic state and may help detect drug-induced redox-epigenetic shifts. If so, simultaneous monitoring of m^5^C and 8-oxo-dG levels in DNA from cancer cells exposed to different drugs may provide useful information on their mechanisms of action [19,20,21].

Glioblastoma (GBM) is a primary astrocytic brain tumor of the central nervous system (CNS) [22]. It is characterized by uncontrolled proliferation, active angiogenesis, invasiveness, and evasion of apoptosis. The median progression-free survival is approximately 6.9 months, and the 5-year survival rate from diagnosis is 9.8% [23,24,25]. Half of the patients die within 2 years of surgery due to tumor recurrence [26]. GBM is biologically complex and difficult to cure. Standard treatment involves maximal surgical resection followed by radiotherapy combined with chemotherapy using the alkylating agent temozolomide (TMZ) [23,24]. However, complete resection is often not possible due to the tumor’s diffuse infiltrative nature, and therefore recurrence is frequent. Despite progress in diagnostics and treatment, the prognosis of patients with glioblastoma remains poor.

Current therapeutic strategies may only prolong survival and improve quality of life, but they do not cure the disease. Chemotherapy remains an important option for treatment. However, drug penetration into the CNS may be limited by the blood–brain barrier (BBB). Moreover, resistance to existing therapies and tumor relapse are commonly observed. TMZ resistance may involve multiple mechanisms, including enhanced DNA repair, MGMT-mediated removal of O6-methylguanine lesions, mismatch repair alterations, changes in apoptosis, drug efflux, cancer stem-like cell populations, metabolic rewiring, and protective signaling pathways [27,28]. Therefore, a better understanding of the molecular mechanisms of action of clinically used drugs is needed to identify new approaches with improved therapeutic potential and acceptable toxicity.

Several currently used drugs, including temozolomide [23,24,29], dexamethasone (DEX) [30,31], valproic acid (VPA) [32,33,34,35], and metformin (MF) [36,37,38,39] may affect glioma biology not only through their primary clinical indications, but also through additional molecular mechanisms. DEX is of particular interest because it is routinely and widely administered during several glioblastoma treatment phases [40,41,42,43,44,45,46].

Patients with GBM usually show peritumoral vasogenic edema and neurological symptoms associated with increased intracranial pressure [47,48,49,50,51]. This edema results from BBB disruption and increased vascular permeability, allowing plasma components to enter the interstitial space [47]. Dexamethasone is routinely used to reduce edema, decrease intracranial pressure, and improve neurological symptoms. It is a synthetic glucocorticosteroid with strong anti-inflammatory, anti-allergic, and immunosuppressive properties [47]. Due to its moderate lipophilicity (log P 1.85) and chemical structure, DEX readily penetrates the BBB, allowing it to act within the CNS. It reduces capillary permeability, and stabilizes the blood–brain barrier [47,52]. Although dexamethasone has been used for brain tumor-related edema for decades, its molecular mechanism remains incompletely understood. Emerging evidence suggests that dexamethasone may also have unfavorable effects in patients with glioblastoma, as longer, higher-dose, or treatment-phase-specific exposure has been associated with shorter overall survival and progression-free survival in several clinical cohorts [30,31,41,42,43]. These studies also showed the role of dexamethasone in patients with clinically significant edema, and support the view that steroid exposure should be carefully justified, minimized when possible, and better understood biologically. The clinical observations also suggest that dexamethasone may interfere with several anticancer treatment modalities, including radiotherapy, chemotherapy, tumor-treating fields, oncolytic viral therapy, and immunotherapy [30,44,45,46]. Therefore, the dose, timing, and cellular consequences of dexamethasone use are important issues in glioblastoma therapy.

Temozolomide is an oral alkylating prodrug that prolongs survival in patients with GBM when administered during and after radiotherapy [23,24,29]. Temozolomide (log P 0.9) readily crosses the blood–brain barrier, and at neutral physiological pH in the bloodstream, undergoes spontaneous conversion to an active intermediate that decays into a methyldiazonium cation. This reactive species exerts its cytotoxic effect mainly through DNA base methylation, predominantly at N7-guanine, N3-adenine, and O6-guanine sites [53]. These lesions interfere with DNA replication and may lead to the apoptosis of cancer cells. The therapeutic response to TMZ is strongly influenced by the methylation status of the MGMT promoter, which regulates the expression of the DNA repair enzyme O6-methylguanine-DNA methyltransferase [54].

Valproic acid is a widely used antiepileptic drug, and seizures are a common presentation in patients with diffuse gliomas. Beyond its antiepileptic activity, VPA has been described as a histone deacetylase inhibitor and may therefore influence chromatin structure and gene expression. It has been investigated as a potential modifier of glioblastoma response to radiotherapy and temozolomide, although clinical results remain heterogeneous [32,33,34]. Recent studies on histone deacetylase inhibitors continue to support the concept that epigenetically active drugs may sensitize glioblastoma models to temozolomide by disturbing cell-cycle progression, reducing viability, limiting proliferation, and inducing apoptosis [35]. Because of its epigenetic activity, valproic acid remains an important compound for studying drug-induced changes in DNA methylation and oxidative DNA damage in glioma cells.

Diabetes is the second most frequent comorbidity in patients with glioblastoma following hypertension. This situation is made worse by the widespread use of dexamethasone, which frequently leads to steroid-related hyperglycemia or diabetes [55,56]. Metformin (log P -2.6) is the most widely prescribed first-line antidiabetic drug globally [57]. It is the antidiabetic drug that GBM patients who have diabetes are most likely to take together with dexamethasone, temozolomide, or valproic acid. Metformin is an amphoteric biguanide that crosses the BBB via organic cation transporters [37]. Metformin has also attracted attention as a potential anticancer agent [36,38,39,58,59]. It inhibits GBM cell growth by activating AMPK, inhibiting mTOR, and modulating oxidative stress [58,59,60,61], including in a concentration-dependent manner, inducing apoptosis in T98G cells [62]. Because metabolism, oxidative stress, and epigenetic regulation are closely connected, metformin may also be relevant in studies of redox-epigenetic mechanisms in glioblastoma [19,63]. In the present study, we included metformin as an additional clinically used metabolic drug in selected viability experiments.

This paper aimed to analyze the epigenetic and redox-related effects of dexamethasone alone and in combination with temozolomide, valproic acid, and metformin in GBM cell lines. We assessed cell viability, real-time proliferation, senescence-associated β-galactosidase activity, cell-cycle distribution, global DNA methylation, and oxidative DNA damage, with particular attention to 5-methylcytosine and 8-oxo-dG as complementary markers of redox-epigenetic remodeling. We hypothesized that DEX may modify glioblastoma cell biology through redox-epigenetic remodeling and may influence the cellular response to TMZ and VPA. A WHO grade 3 astrocytoma line (SW1783) was included alongside T98G to assess whether these effects are dependent on glioma grade.

## 2. Materials and Methods

### 2.1. Chemicals and Reagents

Dexamethasone (No. D4902), temozolomide (No. T2577), metformin hydrochloride (No. PHR1084), and thiazolyl blue tetrazolium bromide (No. M5655) were purchased from Sigma (St. Louis, MO, USA). Sodium valproate was supplied by Sanofi-Aventis, Gentilly, France. A dexamethasone stock solution of 25 mg/mL in methanol, temozolomide of 23 mg/mL in dimethyl sulfoxide (DMSO, No. D2650, Sigma, St. Louis, MO, USA), metformin hydrochloride of 50 mg/mL in water, and sodium valproate of 100 mg/mL also in water were prepared and used to prepare the required concentration in a complete medium. [γ-^32^P] ATP (6000 Ci/mmol) was purchased from Hartmann Analytic GmbH (No. HP-601, Braunschweig, Germany). T4 polynucleotide kinase (No. EK0032) was purchased from USB Thermo Fisher Scientific, Waltham, MA, USA. Micrococcal nuclease from *Staphylococcus aureus* (No. N3755), spleen phosphodiesterase II from *Bovine spleen* (No. P9041), apyrase from potato (No. A6410), P1 nuclease (RNase P1 from *Penicillium citrinum*, No. N8630), phosphatase alkaline from calf intestine (No. P4978), isobutyric acid (No. 8004721000), cellulose plates (No. 1.05577.0001), inorganic salts, histochemical staining kit (No. CS0030-1KT), and methanol were purchased from Sigma/Merck, Darmstadt, Germany. A Genomic Mini Kit for DNA isolation was supplied by A&A Biotechnology (No. 116-250, Gdynia, Poland).

### 2.2. Cell Line and Culture Conditions

The human glioblastoma cell lines: T98G (ATCC-CRL-1690), U138 (ATCC-HTB-16), U118 (ATCC-HTB-15), anaplastic astrocytoma cell line SW1783 (ATCC-HTB-13), human cervical cancer cell line HeLa (ATCC-CCL-2), and human normal lung fibroblast cell line WI-38 (ATCC-CCL-75) were purchased from ATCC (Manassas, VA, USA). The T98G, U138, HeLa, and WI-38 cell lines were cultured in EMEM (No. 30-2003, ATCC, Manassas, VA, USA). The SW1783 cell line was cultured in Leibovitz’s L-15 medium (No. 30-2008, ATCC, Manassas, VA, USA) and U118 in DMEM (No. 21969-035, Gibco, Thermo Fisher Scientific, Waltham, MA, USA). Each medium was supplemented with 10% (*v*/*v*) fetal bovine serum (FBS, No. 30-2020, ATCC, Manassas, VA, USA) and 10 units/mL penicillin and 10 μg/mL streptomycin (penicillin-streptomycin, Thermo Fisher Scientific: No. 15140122, Waltham, MA, USA). Cells were cultured at 37 °C in humidified air with 5% CO_2_. After 24 h, cells were washed with phosphate-buffered saline (PBS, No. D8537, Sigma, Aldrich/Me, St. Louis, MO, USA), resuspended in a fresh medium, and treated with DEX, TMZ, and VPA alone or in combination.

### 2.3. Cell Viability (MTT Test)

Cell viability was evaluated with a dye-staining method, using 3-(4,5-dimethyl-2-thiazolyl)-2,5-diphenyl-2H tetrazolium bromide (MTT) (No. M2128, Merck, Darmstadt, Germany) [64]. Cells were seeded in 96-well culture plates at a density of 1 × 10^4^ cells/well and cultured in supplemented medium at 37 °C in a 5% CO_2_ atmosphere. The cells were treated with: DEX from 0.0005 to 1000 μM; TMZ from 0.4 to 400 μM; VPA from 20 to 20,000 μM; MF from 0 to 5000 μM for combination DEX with TMZ, VPA, and MF: DEX from 1 to 1000 μM, TMZ from 1 to 100 μM, VPA from 50 to 500 μM, and MF from 0 to 1000 μM.

Combination experiments were performed using increasing DEX concentrations with fixed TMZ concentrations of 1, 30, and 100 μM; VPA concentrations of 50, 350, and 500; and MF concentrations of 60, 500, and 1000 μM. After 24 h, the supernatant was removed, and 100 μL of MTT solution in medium (0.5 mg/mL final concentration of MTT) was added to each well for 2 h. After incubation, the unreacted dye was removed by aspiration. The formazan crystals were dissolved in 100 μL/well DMSO and measured spectrophotometrically in a multi-well Synergy2 plate 347 reader (BioTek Instruments, USA) at a test wavelength of 492 nm and a reference wavelength of 690 nm. The half-maximal inhibitory concentrations (IC_50_) were calculated by fitting experimental values to the sigmoidal bell-shaped equation using GraphPad Prism v5.01 (GraphPad Software, Inc., San Diego, CA, USA). Values represent the means from four independent experiments.

### 2.4. Cell Cycle Analysis by Flow Cytometry

A total of 1.5 × 10^5^ cells were seeded onto 12-well cell culture plates and incubated for 24 h with TMZ, DEX, and VPA, as well as their combinations, as follows: 25 and 100 µM DEX, and with TMZ from 1 to 100 µM; 25 and 100 µM DEX, and with VPA from 50 to 350 µM. Control cells were incubated in a fully supplemented growth medium without these compounds. At the end of the incubation period, cells were harvested with trypsin, centrifuged at 200× *g* for 5 min, and the cell culture medium was removed. The pellet was washed twice with 1 mL PBS (No. P4474, Merck, Darmstadt, Germany), centrifuged as before, and resuspended in 30 μL PBS. Then, 1 mL of ice-cold 75% ethanol (No. 8.18760, Merck, Darmstadt, Germany) was added dropwise with mixing and incubated for 2 h at −20 °C. Before flow cytometry-based cell cycle analysis, fixed cells were stained with propidium iodide (PI, 537057, Sigma, St. Louis, MO, USA). After centrifugation (200× *g* for 5 min), cells were washed twice with 1 mL of PBS and spun down. The pellet was incubated in 100 μL of PBS containing 1% FBS, PI (5 μg), and RNase A (20 μg) (RNASEA, Merck, Darmstadt, Germany) for 30 min at 37 °C in the dark. The PI fluorescence was measured by a FACS Calibur flow cytometer (Becton Dickinson). Data were analyzed by FlowJo™ v10 software. This experiment was performed as a single biological replicate per condition.

### 2.5. Treatment of Cell Lines with Dexamethasone

DEX stock solution was added directly to the culture medium (at 90–95% confluence) to obtain different concentrations (1–500 μM), and cells were incubated for 3, 24, and 48 h. For the control, the cells were treated with H_2_O only. After 3–48 h of DEX treatment, cells were washed with PBS, trypsinized, and collected by centrifugation at 4000 rpm (2900× *g*) for 10 min (centrifuge 5804R from Eppendorf, Hamburg, Germany). The cellular pellets were quickly frozen and stored at −20 °C for DNA isolation.

### 2.6. Treatment of Cell Lines with the Combination of Dexamethasone, Temozolomide, Valproic Acid, and Metformin

Dexamethasone, temozolomide, valproic acid, and metformin stock solutions and their dilutions were added directly to the culture medium to achieve the designed concentration. In the TMZ experiments, the final DMSO concentration in each cell culture was 0.8%. Cell cultures (with 90–95% confluence) were washed with PBS, placed in fresh medium, and treated with DEX only (0–200 μM), TMZ (10, 100, 200 μM), VPA (100, 200, 500 μM), MF (50, 100, 1000 μM), and their combination. The control cells were treated with H_2_O (for DEX, VPA, and MF) and DMSO. For TMZ, control cultures received the corresponding vehicle at the same final concentration as the treated cultures. After 24 h of incubation, the cells were washed with phosphate buffer, trypsinized with 0.05% trypsin-EDTA (No. 15400-054, Gibco, Paisley, UK), and collected by centrifugation at 4000 rpm (2900× *g*) for 10 min (centrifuge 5804R from Eppendorf, Hamburg, Germany). The cellular pellets were quickly frozen and stored at −20 °C for DNA isolation.

### 2.7. Real-Time Proliferation (xCELLigence^®^)

The xCELLigence^®^ system enabled the real-time observation of cell proliferation. In that experiment, E-Plates (No. G039109, ACEA Biosciences, San Diego, CA, USA) were used, whose well bottoms are covered with gold microelectrodes. T98G cells were seeded in 16-well E-Plates (ACEA Biosciences) at a density of 8 × 10^3^ cells/well and grown in the medium supplemented with DEX (100 μM), VPA (350 μM), and TMZ (100 μM), as well as their mixes, at 37 °C under a 5% CO_2_ atmosphere. The system performed impedance measurements at 15-min intervals for 144 h. The results are presented as cell index units. The normalization time point corresponds to 2 h (e.g., 2 h after starting the cell culture). The graphs show the normalized cell index (mean) and SD.

### 2.8. Isolation of DNA

Cells were detached from the culture plate using a trypsin-EDTA solution and centrifuged at 1200 rpm (264× *g*) for 10 min at 4 °C (centrifuge 5804R from Eppendorf, Hamburg, Germany). The cell pellet was washed in PBS and then processed for DNA isolation using a genomic DNA extraction kit (A&A Biotechnology), according to the manufacturer’s protocol. The purity and concentration of the DNA preparations were checked by measuring UV absorbance at 260 and 280 nm. The A_260_/A_280_ ratios ranged from 1.8 to 2.0. DNA was stored at −20 °C for further analysis.

### 2.9. DNA Hydrolysis, Post-Labeling, and Thin-Layer Chromatography (TLC)

DNA (1 μg) was dissolved in succinate buffer (pH 6.0) containing 10 mM CaCl_2_ (No. C5670) and digested with 0.001 units of spleen phosphodiesterase II and 0.02 units of micrococcal nuclease for 5 h at 37 °C. Moreover, 0.17 μg of DNA digest was labeled with 1μCi [γ-^32^P]ATP (6000 Ci/mmol, Hartmann Analytic GmbH) and 1.5 units of T4 polynucleotide kinase (USB, UK) in 10 mM bicine-NaOH pH 9.7 buffer containing 10 mM MgCl_2_ (No. M8266), 10 mM DTT (No. D0632), and 1 mM spermidine (No. 85558). After 0.5 h at 37 °C, apyrase (10 units/mL) in the same buffer was added, and the mixture was incubated for another 0.5 h. The 3′ nucleotide phosphate was cleaved off with 0.2 μg RNase P1 in 500 mM ammonium acetate buffer, pH 4.5. The identification of [γ-^32^P]m^5^dC was performed with two-dimensional thin-layer chromatography on cellulose plates using a solvent system: isobutyric acid/NH_4_OH:H_2_O (66:1:17 *v*/*v*) in the first dimension and 0.2 M sodium phosphate (pH 6.8)-ammonium sulfate (No. A4915)-n-propyl alcohol (No. 1.00997) (100 mL/60 g/2 mL) in the second dimension. Radioactive spot analysis was conducted with the Phosphoimager Typhoon Screen (Pharmacia, Uppsala, Sweden) and ImageQuantTL 10.1 software (GE Healthcare, Chicago, IL, USA). For precise calculations, we used the amount of material in spots corresponding not only to m^5^dC, but also to its degradation products such as dC (cytosine) and dT (thymine). The total m^5^C contents were calculated as R = m^5^dC/(m^5^dC + dC + dT) × 100. Each analysis was performed four times.

### 2.10. Analysis of 8-oxo-dG Contents in DNA

DNA was dissolved in 200 µL of a buffer (pH 5.3) containing 40 mM sodium acetate (No. S8750), and 0.1 mM ZnCl_2_ (No. 208086), then mixed with nuclease P1 solution (30 µg), and incubated for 3 h at 37 °C. Then, 30 µL of 1 M Tris–HCl pH 8.0 and 5 µL of alkaline phosphatase (1.5 units) solution were added, followed by a 1 h incubation at 37 °C. DNA hydrolysate was purified using 10 kDa cut-off filter units (No. 0000157924, Merck, Darmstadt, Germany). The 8-oxo-dG amount in DNA was determined using HPL (Agilent Technologies 1260 Infinity, SantaClara, CA, USA) with two detectors working in series: the 1260 Diode Array Detector and the Coulochem III Electrochemical Detector (ESA Inc., Chelmsford, MA, USA). The isocratic chromatography of DNA hydrolysate was performed using a solution of 50 mM CH_3_COONH_4_ at pH 5.3 and methanol (93:7). An analysis of dG for reference was performed at 260 nm. We determined 8-oxo-dG with the following electrochemical detection settings: guard cell +400 mV, detector 1: +130 mV (screening electrode), and detector 2: +350 mV (measuring electrode set to 100 nA sensitivity).

Quantification of the genome amounts of m^5^C and 8-oxo-dG in human DNA was conducted. The number of modified bases in DNA was calculated based on global genome composition.

The amount of modified bases in DNA was calculated on the basis of global genome composition—3.05 × 10^9^ bases (100%), where C-624 × 10^6^ (20.5%), T-905 × 10^6^ (29.6%), G-623 × 10^6^ (20.4%), A-901 × 10^6^ (29.5%), and m^5^C-31 × 10^6^ (1%). The amount of m^5^C (%) in pyrimidines in DNA was determined from TLC analysis with the formula R* [%] = m^5^C × 100/(C + T). The total number of m^5^C in the genome was calculated from the formula m^5^C = (1498,333,975) × R*/100. The input amount of guanosine was necessary to determine the 8-oxo-dG content and was calculated from the Diode Array Detector (DAD) measurements using the Avogadro number NG = 6.02 × 1020 × b(mAU)/a(mAU) standard. The 8-oxo-dG nucleoside amount was estimated with an electrochemical detector with the N8-oxo-dG = 6.02 × 10^20^ × d(nA)/c (nA) standard. The total number of 8-oxo-dG = 623 × 10^6^ × N8-oxo-dG/NG [65].

### 2.11. Imaging Flow Cytometry Analysis of SA-β-gal Activity

Cells were harvested from a confluent T75 flask, and 1 × 10^6^ cells were seeded into T25 flasks. DEX (4 mg/mL) was added to the culture medium to achieve a final concentration of 35 μM at 0 h. After 24 h, an equal amount of DEX was added to refresh the treatment, resulting in a cumulative DEX concentration of 70 μM. At 48 h, cells were trypsinized and centrifuged at 1500 g for 5 min. The cell pellet was resuspended in 100 µL of 4% formaldehyde (No. F8775) and fixed for 10 min at room temperature. Fixed cells were centrifuged, washed twice with PBS, and stained for SA-β-gal activity using the CellEvent™ Senescence Green Detection Kit (Thermo Fisher Scientific) for 2 h, following the manufacturer’s protocol, and subsequently counterstained with NucSpot™ 650 (Biotium) for DNA visualization. Samples were analyzed on an Amnis^®^ ImageStream^®^ imaging flow cytometer (Luminex). We excited fluorescence using 488 nm and 642 nm lasers. Gating was applied based on cell area and aspect ratio to exclude debris and doublets. This experiment was performed as a single biological replicate, with 500 cell images acquired per condition. Image analysis, including overlays, histograms, and statistical measurements, was performed using IDEAS^®^ software 15.15 (Bratislava Slovakia, Luminex).

### 2.12. Statistical Analysis

Microsoft Excel 2010, with its data analysis package, and GraphPad Prism v5.01 were used to analyze all data. Unless stated otherwise, data resulted from four independent experiments. The cell-cycle assay (Section 2.4) and SA-β-gal assay (Section 2.11) were performed as a single experiment. The descriptive statistics function generated the mean and SD, and the results were shown as error bars. A two-tailed *t*-test was used to calculate significant differences in m^5^C content for the tested samples compared to the control experiments, and a *p*-value < 0.05 was considered significant.

## 3. Results

### 3.1. Cytotoxic Effects of DEX, TMZ, VPA, and MF in T98G Glioblastoma Cells

For the initial assessment of drug-induced cytotoxicity, we selected the T98G glioblastoma cell line. T98G is IDH1/2-wildtype [66], consistent with the diagnostic category “glioblastoma, IDH-wildtype” that the current WHO classification uses for the large majority of adult primary glioblastomas [22]. It also carries an activating TERT promoter mutation [67]. Together with its established treatment-resistant phenotype, including temozolomide resistance associated with MGMT expression and DNA repair capacity [68,69,70], these features made T98G particularly suitable for testing whether DEX or MF modifies treatment response against a resistant molecular background. Cell viability was assessed using the MTT assay, a colorimetric method commonly applied to evaluate cell metabolic activity and survival after drug exposure [64].

T98G cells were treated for 24 h with increasing concentrations of DEX, TMZ, and MF in the range of 1–5000 µM (0–3.6 on a logarithmic scale), and VPA in the range of 0–10,000 µM (0–4 on a logarithmic scale), either alone or in selected combinations. The results are presented on a logarithmic concentration scale (Figure 1). At low concentrations, up to approximately 60 µM (1.8 on a logarithmic scale), DEX, TMZ, VPA, and MF had comparable and relatively limited effects on T98G cell viability. Among the compounds tested individually, VPA showed the lowest cytotoxicity, but DEX was most toxic. MF displayed low cytotoxicity under the tested conditions (Figure 1).

These cytotoxicity findings are specific to T98G. DEX cytotoxicity varies substantially across glioma cell lines [71], so we do not extend these viability conclusions to U138, U118, or SW1783 without direct testing. These observations suggest that DEX may modify the cellular response of T98G glioblastoma cells to clinically relevant co-administered drugs. Cell viability after treatment with a mixture of DEX with TMZ and DEX with VPA remained at the same level for low DEX concentrations up to 60 µM and varied at TMZ concentrations from 1 to 30 µM and VPA concentrations of 50 µM. At TMZ concentrations above 100 µM and VPA at 350 µM, viability decreased by 30–40% (Figure 1B,C). Thus, the toxicity of the DEX and VPA mixture is higher than that of DEX and TMZ, and VPA/TMZ and MF/TMZ. Interestingly, DEX in a wide range of concentrations does not affect MF treatment.

### 3.2. Real-Time Proliferation of T98G Cells After DEX, TMZ, and VPA Treatment

We next evaluated the effects of DEX and other drug combinations on cell proliferation. We monitored the real-time proliferation of T98G glioblastoma cells using the xCELLigence system, which allows for continuous impedance-based assessment of cell number, adhesion, and morphology. Changes in electrical impedance are expressed as the cell index and provide dynamic information on cellular responses to drug exposure [72,73].

T98G cells were seeded on E-Plates containing gold microelectrodes and cultured in complete medium supplemented with DEX (100 µM), TMZ (100 µM), VPA (350 µM), or selected drug combinations. Untreated cells cultured in complete medium served as the control. Impedance was recorded continuously for 144 h, and 12 time points are shown as normalized cell index values over time (Figure 2). During 48 h, T98G cells showed comparable proliferation kinetics under all tested conditions, with no statistically significant differences between the control and drug-treated groups. After 48 h, the inhibitory effects of the tested compounds became more apparent. Cells treated with DEX alone and cells exposed to DEX-containing combinations with VPA and TMZ showed a marked decrease in normalized cell index. This decrease is consistent with reduced proliferation and/or loss of viable adherent cells rather than direct proof of a specific mechanism of cell death. In contrast, cells treated with TMZ or VPA alone maintained a higher cell index over time, indicating less pronounced inhibition of proliferation. The TMZ/VPA combination also did not reduce the cell index to the same extent as the DEX-containing treatments. Overall, these results indicate that DEX exerted the strongest inhibitory effect on T98G cell proliferation. The data also suggest that the TMZ/VPA combination may partially attenuate the decrease in cell index observed after DEX exposure. However, DEX combined with TMZ and VPA slightly increased cell proliferation.

### 3.3. Effects of DEX and VPA on SA-β-gal Activity

Therapy-induced senescence is increasingly recognized as a complex cellular response to anticancer treatment. Although senescence is classically associated with stable cell-cycle arrest, senescent cancer cells may also contribute to tumor progression through senescence-associated secretory phenotype, cellular plasticity, and in selected contexts, escape from senescence-associated growth arrest [74,75]. Therefore, an evaluation of senescence-related markers may provide additional information on the cellular response to drug exposure. Senescence-associated β-galactosidase (SA-β-gal) activity is one of the most widely used biomarkers of cellular senescence and reflects increased lysosomal β-galactosidase activity detectable under assay-specific conditions [76,77]. In the present study, we assessed SA-β-gal activity in WI-38 fibroblasts and T98G glioblastoma cells after DEX treatment using a fluorogenic β-galactosidase substrate (Figure 3). WI-38 and T98G cells were treated with DEX for 48 h and compared with the untreated controls. DEX-treated WI-38 cells showed a 1.32-fold increase in the mean SA-β-gal activity of the cell population compared with the control, consistent with the induction of a senescence-like phenotype in normal fibroblasts. In contrast, DEX-treated T98G cells exhibited a 1.57-fold decrease in the mean SA-β-gal activity relative to the control. This divergent response suggests that DEX affects SA-β-gal activity in a cell-type-dependent manner. In T98G cells, the decrease in SA-β-gal signal may reflect reduced metabolic activity, altered lysosomal function, or the loss of viable adherent cells rather than the classical induction of senescence. Because SA-β-gal activity is a widely used but not fully specific marker of cellular senescence, these findings should be interpreted together with morphological changes, proliferation data, and markers of oxidative DNA damage [78].

### 3.4. Effects of DEX, TMZ, and VPA on Cell-Cycle

To assess whether the tested compounds affected cell-cycle progression, T98G, SW1783, and WI-38 cells were treated with DEX, TMZ, and VPA, either alone or in selected combinations, and analyzed by flow cytometry after propidium iodide staining (Figure 4). Cell-cycle analysis based on DNA content is a widely used approach for evaluating drug-induced changes in the distribution of cells between the G0/G1, S, and G2/M phases [79].

In the WI-38 and SW1783 cells, treatment with DEX, TMZ, VPA, or their combinations did not induce marked changes in cell-cycle distribution under the experimental conditions used. In contrast, T98G glioblastoma cells showed a more evident response. Treatment with 100 µM TMZ, both alone and in combination with DEX, increased the proportion of cells in the G1 phase. This effect was not observed after DEX alone or after DEX combined with VPA.

The TMZ-associated shift in the T98G cell cycle is consistent with the activation of DNA damage response pathways following alkylating treatment. TMZ induces DNA methylation lesions, including N7-methylguanine, N3-methyladenine, and O6-methylguanine, which may interfere with DNA replication and activate checkpoint mechanisms depending on the cell line’s repair capacity and molecular background [53,80]. Therefore, the increased G1 fraction observed in T98G cells may reflect TMZ-induced cell-cycle perturbation rather than a direct effect of DEX. The lack of a comparable effect in DEX- or VPA-treated cells suggests that under these conditions, TMZ was the main driver of cell-cycle changes.

### 3.5. Effects of DEX on Global DNA Methylation in Glioma Cell Lines

The effect of DEX on global DNA methylation was assessed by measuring the total 5-methylcytosine content, expressed as the R value, in four glioma cell lines, one cancer cell line, and one normal human fibroblast cell line. Cells were treated with increasing concentrations of DEX ranging from 1 to 500 µM for 3, 24, and 48 h (Figure 5). DEX induced concentration- and time-dependent changes in global DNA methylation. In the glioblastoma cell lines T98G, U138, and U118, DEX exposure was associated with increased m^5^C content, particularly after shorter incubation times. In the T98G cells, DEX induced a significant increase in global DNA methylation at several tested concentrations up to 24 h. However, after 48 h of continuous exposure, m^5^C levels decreased relative to earlier time points, suggesting dynamic remodeling of DNA methylation during longer DEX treatment. SW1783 cells, an astrocytic glioma-derived cell line, showed a different pattern, with the increased methylation level after 48 h remaining relatively stable. These findings indicate that DEX’s effect on global DNA methylation is both time-dependent and cell-line specific. The observed differences between glioblastoma-derived and astrocytic glioma-derived cell lines may reflect distinct molecular backgrounds, stress responses, and epigenetic plasticity.

The T98G cell line, which is the best glioma counterpart, has an IDHwt phenotype, is more aggressive and treatment-resistant with respect to MGMT promoter methylation, and shows a significant increase in DNA methylation at all concentrations up to 24 h. Longer incubation time (48 h) showed decreased m^5^C levels, suggesting demethylation (Figure 5). For the HeLa cell line, the highest increase was observed at 3 h, for the non-tumor cell line at 3 and 24 h, followed by slow demethylation.

### 3.6. Effects of DEX/TMZ/VPA/MF Combinations on Global DNA Methylation in T98G

The effects of DEX, TMZ, VPA, and MF on global DNA methylation were analyzed in T98G cells. Total m^5^C content was assessed after 24 h treatment with DEX at concentrations ranging from 0 to 200 µM, either alone or in combination with TMZ at 0–200 µM, VPA at 0–500 µM, and MF at 0–1000 μM (Figure 6A–C).

TMZ, VPA, and MF-containing treatment conditions modified global m^5^C levels in a concentration-dependent manner. Lower DEX concentrations, particularly 25 µM, were associated with increased m^5^C content when combined with TMZ, VPA, and MF. DEX at 25, 50, and 100 µM did not markedly reduce the TMZ-associated increase in DNA methylation. Demethylation was observed only at 200 µM. In combination with VPA, m^5^C levels decreased at a DEX concentration of 50 µM.

Metformin alone induced DNA demethylation, which was reversed at a DEX concentration of 25 μM (Figure 6C). Overall, these results indicate that the effects of DEX, TMZ, VPA, and MF on global DNA methylation are not uniform, but depend on the drug concentrations and their combination. Low concentrations of DEX, particularly in combination with TMZ, were generally associated with increased m^5^C content, whereas higher concentrations of DEX tended to reduce global DNA methylation. Low concentrations of DEX increase DNA methylation.

### 3.7. Analysis of 8-oxo-dG in DNA of the T98G and SW1783 Cell Lines Treated with DEX, TMZ, and VPA

The effects of the three drugs, alone and in combination, on the total content of 8-oxo-dG in DNA isolated from the T98G and SW1783 cell lines were analyzed. The 8-oxo-dG levels decreased in both cell lines. DEX at 25–100 µM in the T98G cells and 25–50 µM in the SW1783 cells, in the presence of TMZ, decreased the 8-oxo-dG levels (Figure 7A–C). On the other hand, a high concentration of DEX (100 and 200 µM) in the case of SW1783 induced an increase in 8-oxo-dG content (Figure 7C). However, the combination of DEX with VPA in the T98G cell line for the entire range of DEX concentrations decreased 8-oxo-dG content (Figure 7B). On the other hand, for SW1783, we observed an increase in 8-oxo-dG for DEX concentrations of 25–200 µM (Figure 7D). A general decrease in 8-oxo-dG suggests the antioxidant effect of DEX as well as the induction of DNA repair mechanisms.

### 3.8. Analysis of m^5^C and 8-oxo-dG in DNA of the T98G Cell Lines Treated with DEX and Different Ratios of TMZ/VPA

To evaluate the combined epigenetic and oxidative effects of DEX and TMZ/VPA treatment, T98G cells were incubated for 24 h with DEX at concentrations ranging from 50 to 200 µM and with TMZ/VPA mixtures corresponding to TMZ-to-VPA ratios ranging from 0 to 0.4 (Figure 8). Combined treatment generally decreased the global m^5^C content in DNA, indicating a shift toward DNA demethylation. This effect was accompanied by a reduction in 8-oxo-dG content. Thus, the decrease in global DNA methylation was not associated with increased oxidative DNA damage. Instead, combined exposure to DEX and TMZ/VPA produced parallel reductions in both m^5^C and 8-oxo-dG levels, although the magnitude of these changes varied by DEX concentration and TMZ/VPA combination.

## 4. Discussion

This study shows that DEX induces dynamic, concentration-, time-, and cell-line-dependent changes in global DNA methylation and oxidative DNA damage, and modifies glioma-cell responses to TMZ, VPA, and MF.

We evaluated DEX alone and in combination with TMZ or VPA, and included MF in selected cell-viability experiments as an additional clinically used metabolic drug. This experimental design reflects the complexity of glioblastoma management, in which tumor-directed treatment, antiepileptic therapy, corticosteroid administration for peritumoral edema, and medications used for comorbid conditions may overlap during the course of disease [31,41].

The study was based on the premise that the drugs, although developed primarily as non-cancer agents, may modify glioblastoma cell behavior through redox–epigenetic responses. DNA methylation is a key epigenetic mechanism involved in genome regulation, chromatin organization, cellular differentiation, and gene-expression control. In cancer, global DNA hypomethylation together with locus-specific promoter hypermethylation is always observed and may contribute to genomic instability, altered transcriptional programs, tumor progression, and treatment resistance [3,16]. In GBM, the clinical relevance of DNA methylation is well-illustrated by the predictive role of MGMT promoter methylation in determining response to TMZ [54]. Global m^5^C content, however, reflects a broader dimension of the epigenetic state and may capture treatment-related changes in cellular adaptation, stress responses, and epigenetic plasticity. Its biological significance can be inferred only from the direction of change, although global measurements do not identify the genomic regions where methylation is gained or lost.

We previously showed that TMZ may act not only as a cytotoxic alkylating agent, but also as an agent that modifies global DNA methylation in glioma cells [20,21]. We also reported that changes in total DNA methylation may be associated with oxidative DNA damage, supporting the concept of redox-linked epigenetic remodeling in gliomas [20,21]. In the present work, we extended this approach to DEX and its combinations with TMZ, VPA, and MF. We assessed m^5^C and 8-oxo-dG as complementary markers of global DNA methylation and oxidative DNA damage. Although we studied MF in selected viability experiments, we interpreted it mechanistically in relation to DNA methylation or oxidative DNA damage.

A major observation was that DEX induced concentration- and time-dependent changes in global DNA methylation. In the GBM-derived T98G, U118, and U138 cell lines, shorter DEX exposure generally increased the m^5^C content. In the T98G cells, this increase remained evident for up to 24 h, whereas after prolonged exposure, the m^5^C levels decreased relative to the earlier time points. These findings suggest that the DEX-associated epigenetic response is dynamic and may evolve during continued exposure rather than representing a stable unidirectional effect.

The response to DEX was cell-line dependent. T98G cells, a widely used glioblastoma-derived model characterized by relative resistance to TMZ, showed a marked but time-dependent methylation response [68,69]. SW1783 cells showed a distinct pattern and appeared more responsive to high-dose DEX in the combination experiments. These differences may reflect variation in molecular background, basal redox state, DNA repair capacity, glucocorticoid responsiveness, and epigenetic plasticity [81,82,83,84]. The findings are therefore consistent with the well-recognized heterogeneity of glioma models and indicate that the biological effects of DEX cannot be generalized across all glioma cell types.

The combination experiments showed that DEX modifies the methylation response associated with TMZ. Low DEX concentrations, particularly 25–50 µM, were generally associated with increased m^5^C content in TMZ-containing conditions. In the T98G cells, low to moderate DEX concentrations did not markedly suppress the TMZ-associated increase in global DNA methylation. However, in the SW1783 cells, higher DEX concentrations were associated with reduced m^5^C levels, indicating a shift toward global DNA hypomethylation. The VPA-containing conditions showed a distinct pattern. VPA is clinically relevant in glioblastoma because seizures are common in patients with diffuse gliomas and is also biologically relevant because of its histone deacetylase-inhibitory activity. VPA may influence chromatin structure, gene expression, cell-cycle regulation, and treatment response, although clinical data remain heterogeneous [32,33,34,35]. In the present study, VPA combined with low-dose DEX increased the m^5^C content in T98G cells, whereas higher DEX concentrations shifted the response toward reduced global methylation. In the SW1783 cells, VPA-containing conditions were associated with decreased global DNA methylation across the analyzed DEX concentrations. Genome-wide hypomethylation is a common feature of cancer, and given that an experimentally induced increase in total m^5^C is beneficial, a reduction indicates a uniformly adverse effect. We evaluated oxidative DNA damage by measuring 8-oxo-dG, a widely used marker of guanine oxidation and oxidative stress-related DNA damage [85]. In the T98G cells, TMZ- and VPA-containing conditions were generally associated with low 8-oxo-dG levels, and the addition of DEX at concentrations of up to 100 µM did not markedly increase guanine oxidation. In contrast, the SW1783 cells showed increased 8-oxo-dG together with reduced global m^5^C after exposure to higher DEX concentrations, particularly in TMZ-containing conditions. This pattern suggests that high-dose DEX may shift SW1783 cells toward oxidative DNA damage accompanied by global DNA hypomethylation.

The decrease in 8-oxo-dG observed in several T98G treatment conditions may reflect reduced lesion formation, enhanced repair or removal of oxidized guanine, altered cellular metabolism, changes in nucleotide-pool sanitation, or the selective survival of less damaged cells.

The combined assessment of m^5^C and 8-oxo-dG in the T98G cells treated with DEX and different TMZ/VPA ratios provided further insight into the relationship between DNA methylation and oxidative damage. Under these conditions, global DNA hypomethylation was accompanied by reduced 8-oxo-dG levels. Thus, in this model, reduced m^5^C levels were not necessarily associated with increased oxidative guanine damage. This decoupling likely reflects the fact that DNA methylation is a tightly enzymatically regulated process catalyzed by DNA methyltransferases [1,2], whereas oxidative DNA damage arises through the comparatively stochastic direct reaction of guanine with reactive oxygen species [17], allowing the two markers to diverge under combined treatment. In the MTT assay, DEX/TMZ and DEX/VPA combinations reduced T98G cell viability in a concentration-dependent manner, with a more pronounced reduction after DEX/VPA than after DEX/TMZ under the conditions tested. MF displayed limited cytotoxicity in the selected viability experiments. This finding should be interpreted in relation to the tested concentrations and exposure time as well as MF’s effects on global DNA methylation or oxidative DNA damage.

Real-time xCELLigence analysis showed that DEX produced the strongest delayed reduction in the T98G cell index, particularly after 48 h. Because the xCELLigence system measures impedance, the decline in cell index may reflect reduced proliferation, altered adhesion or morphology, and/or loss of viable adherent cells. The cell-cycle findings further distinguished the effects of the tested drugs. TMZ, rather than DEX or VPA, was the principal compound associated with cell-cycle redistribution in T98G cells. Treatment with 100 µM TMZ increased the proportion of cells in the G1 phase, both alone and in combination with DEX. This observation is consistent with TMZ-induced DNA alkylation, activation of DNA damage responses, and checkpoint signaling [28,80]. The lack of a comparable change after DEX alone or DEX/VPA treatment indicates that the observed cell-cycle effect was primarily associated with TMZ under the experimental conditions used.

The senescence-related response is also cell-type dependent. DEX increased SA-β-gal activity in WI-38 fibroblasts, consistent with a senescence-like response in normal fibroblasts. We included WI-38, among other reasons, because fibroblasts and connective tissue are well-documented off-target sites of systemic glucocorticoid action, making this line a physiologically relevant reference beyond the CNS. Our findings are consistent with a previous study demonstrating that DEX induces senescence-associated changes in trabecular meshwork cells by increasing ROS [86]. In contrast, DEX-treated T98G cells showed reduced SA-β-gal fluorescence. Because SA-β-gal reflects increased lysosomal β-galactosidase activity and is not fully specific for cellular senescence, the reduced signal in T98G cells should not be interpreted simply as absence of senescence or as direct evidence of cell death [76,77,78]. It may instead reflect reduced viability, altered lysosomal function, metabolic changes, or loss of adherent cells. This distinction matters because therapy-induced senescence in cancer may have complex, context-dependent consequences, including the development of a senescence-associated secretory phenotype, increased plasticity, and, in selected settings, escape from growth arrest and the acquisition of stem-like features [74,75].

DEX has substantial clinical relevance in glioblastoma. DEX remains essential for the symptomatic management of peritumoral edema and increased intracranial pressure. At the same time, experimental and clinical evidence indicates that corticosteroid exposure may be associated with less favorable outcomes in patients with glioblastoma. Experimental studies have suggested that corticosteroids may compromise survival in glioblastoma models and patients [30], while other meta-analyses have reported associations between DEX exposure and worse overall and progression-free survival [30,31,41,42,43]. These observations do not diminish the clinical necessity of DEX in symptomatic patients. Rather, they support the careful justification of steroid therapy and the use of the lowest effective dose for the shortest clinically appropriate duration.

Although the present in vitro findings cannot directly explain the clinical associations between corticosteroid exposure and outcome, they support the concept that DEX is biologically active in glioma cells. Beyond its established anti-edematous and anti-inflammatory effects, DEX influenced the global DNA methylation levels, oxidative DNA damage, viability, real-time cell-index kinetics, and senescence-associated activity, and modified cellular responses to TMZ and VPA. From a drug-repurposing and supportive-care perspective, this is important because medications routinely administered during glioblastoma treatment may alter tumor-cell biology even when they are not intended to act directly against the tumor.

The present study provides evidence that DEX modifies glioma-cell redox–epigenetic responses and alters cellular responses to TMZ and VPA, whereas MF showed limited cytotoxicity in the selected viability experiments. The parallel assessment of m^5^C and 8-oxo-dG identified treatment conditions associated with global DNA hypermethylation, hypomethylation, and oxidative guanine damage and showed that these changes are not uniformly coupled. This approach may provide a useful basis for future mechanistic studies examining how supportive and repurposed drugs influence glioblastoma-cell adaptation, resistance, and response to treatment.

These findings may have translational implications beyond DEX itself. We have previously proposed that global DNA methylation approaching that of healthy tissue, a state we term eumethylation, represents a potential therapeutic target in glioma, since methylation progressively declines with disease severity and malignancy grade [87]. The present and prior data [20,88] indicate that hypermethylating effects are more readily achieved with lower, and potentially repeated, drug exposures than with single high doses, which instead tend to induce demethylation. This raises the possibility of adjusting dosing regimens to favor lower, more frequent administration to shift glioma cells toward a more favorable epigenetic state, rather than relying solely on maximal cytotoxic dosing. Because global DNA methylation can be assessed in peripheral blood with minimally invasive, low-cost assays, this endpoint could, in principle, be monitored alongside drug-concentration measurements in future clinical studies and might also serve as a screening criterion for identifying additional methylation-modifying agents suitable for co-therapy with DEX, TMZ, or VPA.

The concentration ranges tested here vary in their relationship to clinically achievable exposure. Brain or CSF concentrations reported for DEX, TMZ, and MF are one to three orders of magnitude lower than the higher end of the ranges tested in the present study [53,58,89]. In contrast, our previous work explicitly anchored TMZ and VPA concentrations to levels reached in the brain during treatment [88,90], and the VPA concentrations used in the present combination experiments (50–350 µM) fall within this clinically relevant range. Similarly, our earlier characterization of MF included concentrations spanning its known brain-penetrant range (1–100 µM) [19]. Here, we intentionally extended DEX, TMZ, and MF exposure beyond these levels to characterize their concentration-dependent effects more broadly; findings at the higher end of these ranges should accordingly be interpreted as mechanistic rather than directly reflecting standard clinical dosing, whereas the VPA findings and the lower end of the MF range correspond to clinically achievable exposure. Standard culture conditions (≈18–21% O_2_) are considerably more oxygenated than normal brain tissue (physioxia, ~2–6% O_2_) or glioblastoma tissue itself, which is frequently severely hypoxic (median tumor pO_2_ ~10 mmHg) [91,92].

We performed the drug-combination experiments, viability assays, real-time proliferation assays, and cell-cycle analysis in T98G cells. Conclusions drawn from these experiments are therefore specific to this line and should not be extended to glioblastoma more broadly without confirmation in additional IDH–wildtype lines or patient-derived models. In contrast, we performed global DNA methylation and 8-oxo-dG analyses across four glioma lines, along with HeLa and WI-38.

## 5. Conclusions

DEX induced concentration-, time-, and cell-line-dependent changes in global DNA methylation and oxidative DNA damage in glioma cells. In the T98G cells, shorter DEX exposure generally increased the m^5^C content, whereas prolonged exposure and selected combination treatments shifted the response toward reduced global methylation. In the SW1783 cells, high DEX concentrations were associated with reduced m^5^C and increased 8-oxo-dG. DEX also modified the methylation and oxidative-damage responses to TMZ and VPA and produced a delayed reduction in the xCELLigence cell index. TMZ, rather than DEX or VPA, was the principal compound associated with cell-cycle redistribution in the T98G cells.

The parallel assessment of m^5^C and 8-oxo-dG indicates a complex, context-dependent relationship between global DNA methylation and oxidative guanine damage.

Overall, the findings support the view that DEX is biologically active in glioma cells and may modify their redox–epigenetic state and response to co-administered drugs.

## Figures and Tables

**Figure 1:**
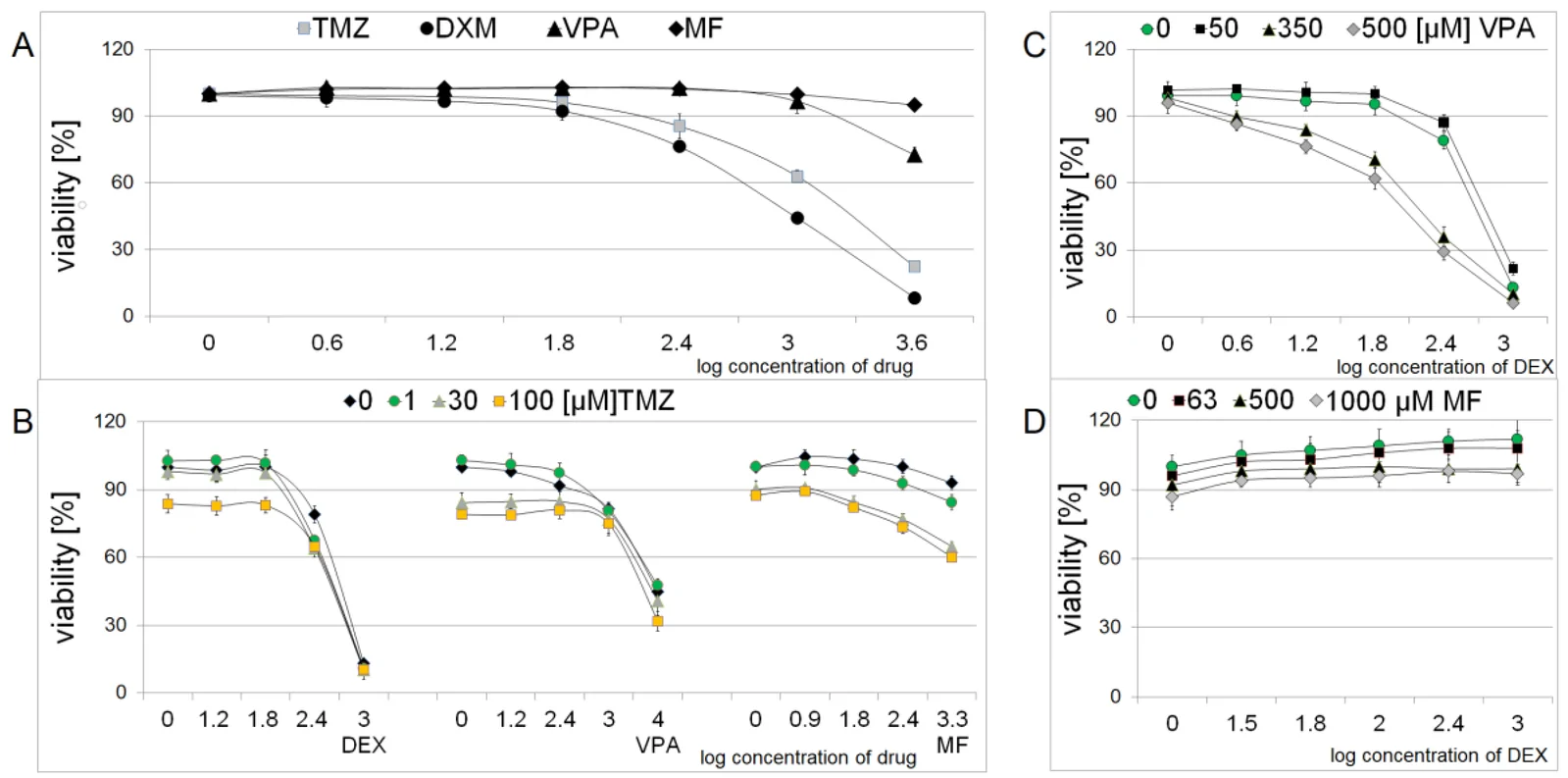
Effects of dexamethasone (DEX), temozolomide (TMZ), valproic acid (VPA), and metformin (MF) on T98G glioblastoma cell viability assessed by the MTT assay (A), and their combination with TMZ (B), and treatment with DEX and VPA (C), and DEX and MF (D), on the cell viability of cell line T98G. Cells were treated for 24 h with increasing concentrations of the tested drugs, either alone or in selected combinations. Data are presented as mean cell viability ±SE from four independent experiments. Statistical comparisons were performed using two-way ANOVA followed by Tukey’s multiple-comparisons test, as detailed in Supplementary Table S1.

**Figure 2:**
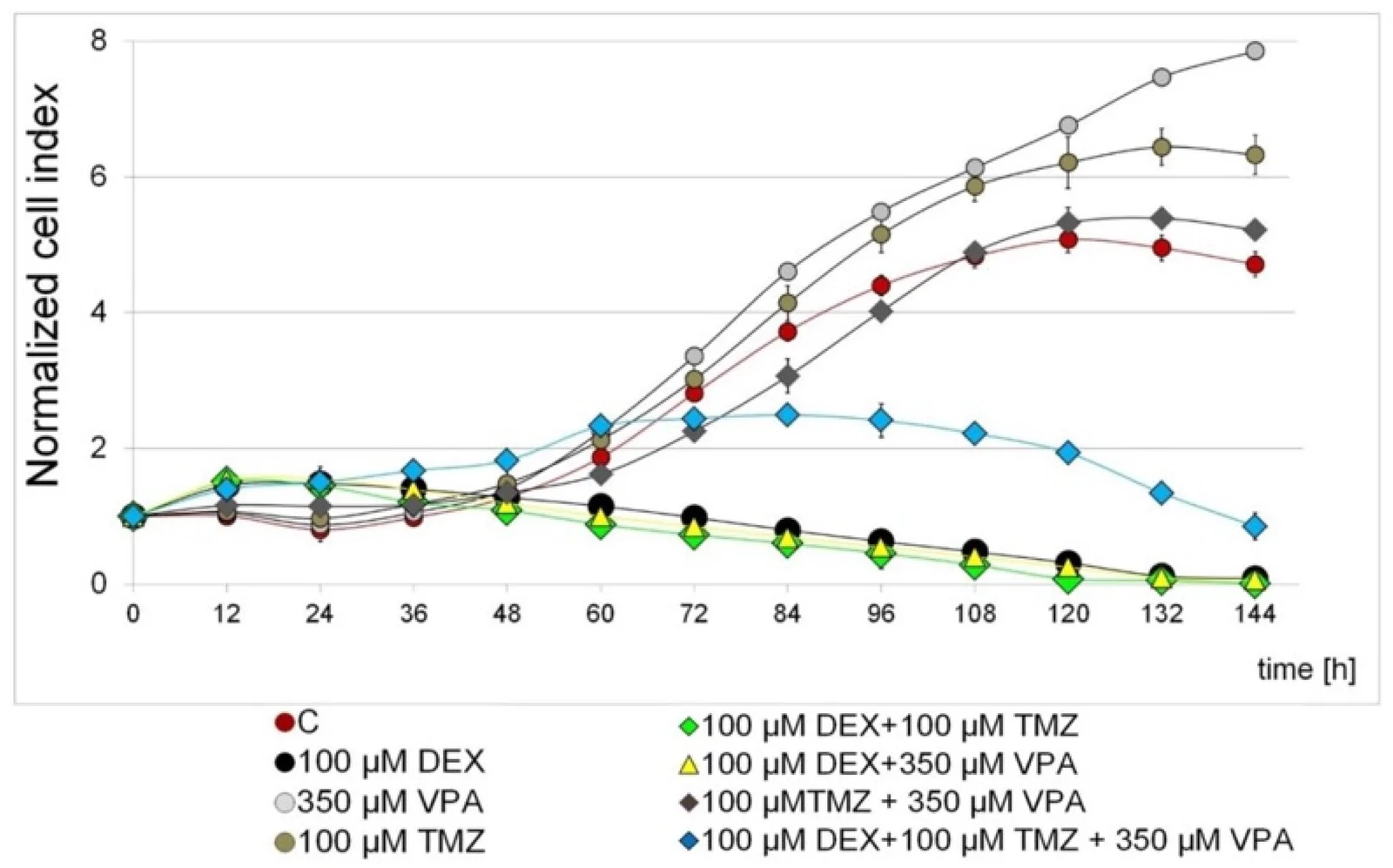
Real-time proliferation of T98G glioblastoma cells after treatment with DEX, TMZ, VPA, and their selected combinations. We monitored cell proliferation using the xCELLigence system for 144 h and expressed it as a normalized cell index. Control cells were cultured in complete medium without the tested compounds. Data are presented as mean ± SD. Differences between the treatment groups and the corresponding controls were assessed using two-tailed paired *t*-tests. DEX-containing treatments showed the strongest delayed decrease in cell index after 48 h. The statistical analysis is presented in Supplementary Table S2.

**Figure 3:**
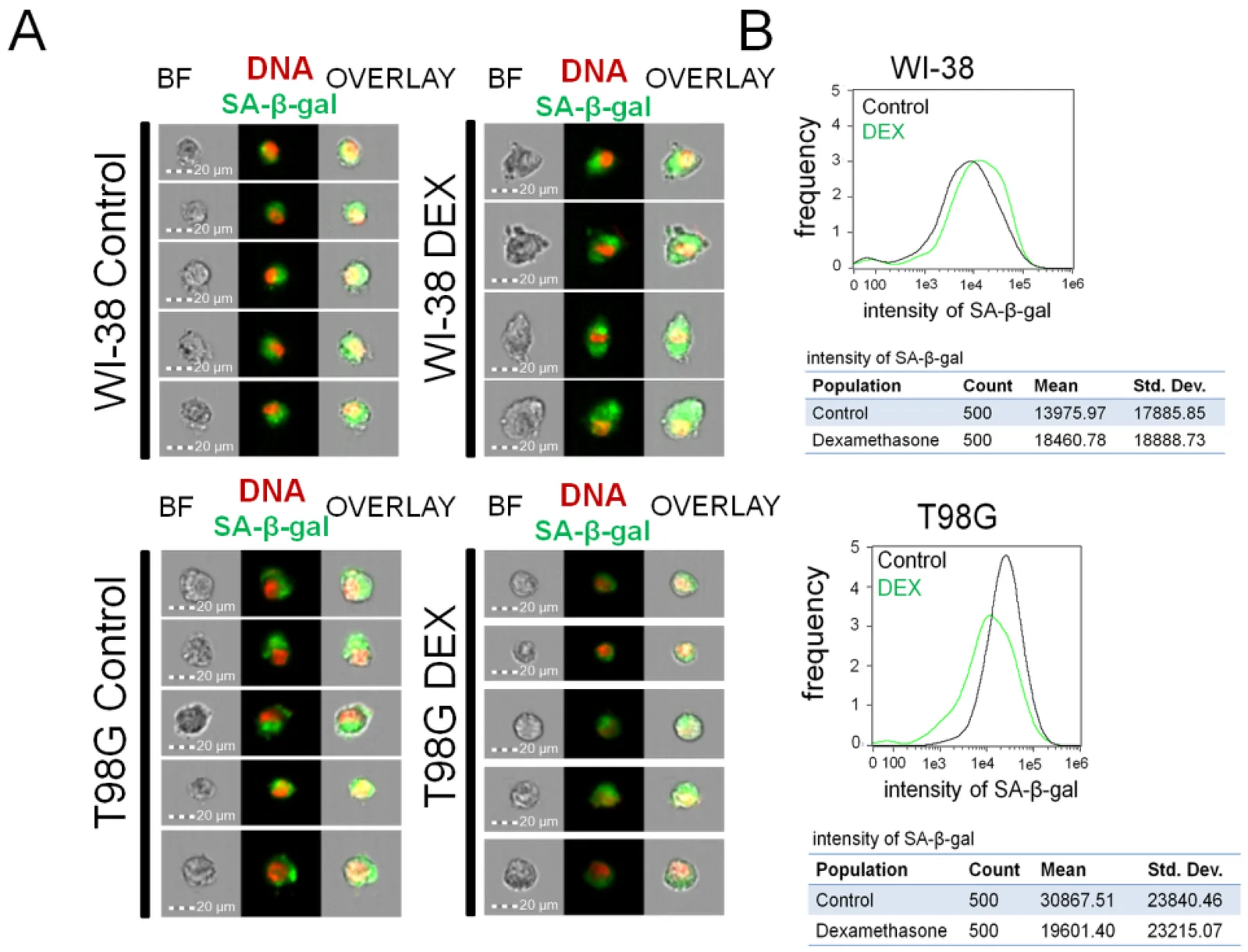
Imaging flow cytometry analysis of SA-β-gal activity in the WI-38 and T98G cell lines following 48 h dexamethasone treatment (**A**). Representative images of control and dexamethasone-treated cells on channels: bright field (BF), DNA and SA-β-gal staining, and an overlay of all channels together (**B**). Histograms showing SA-β-gal fluorescence intensity for 500 cells per condition. Data derived from a single experiment—no formal statistical comparison was performed.

**Figure 4:**
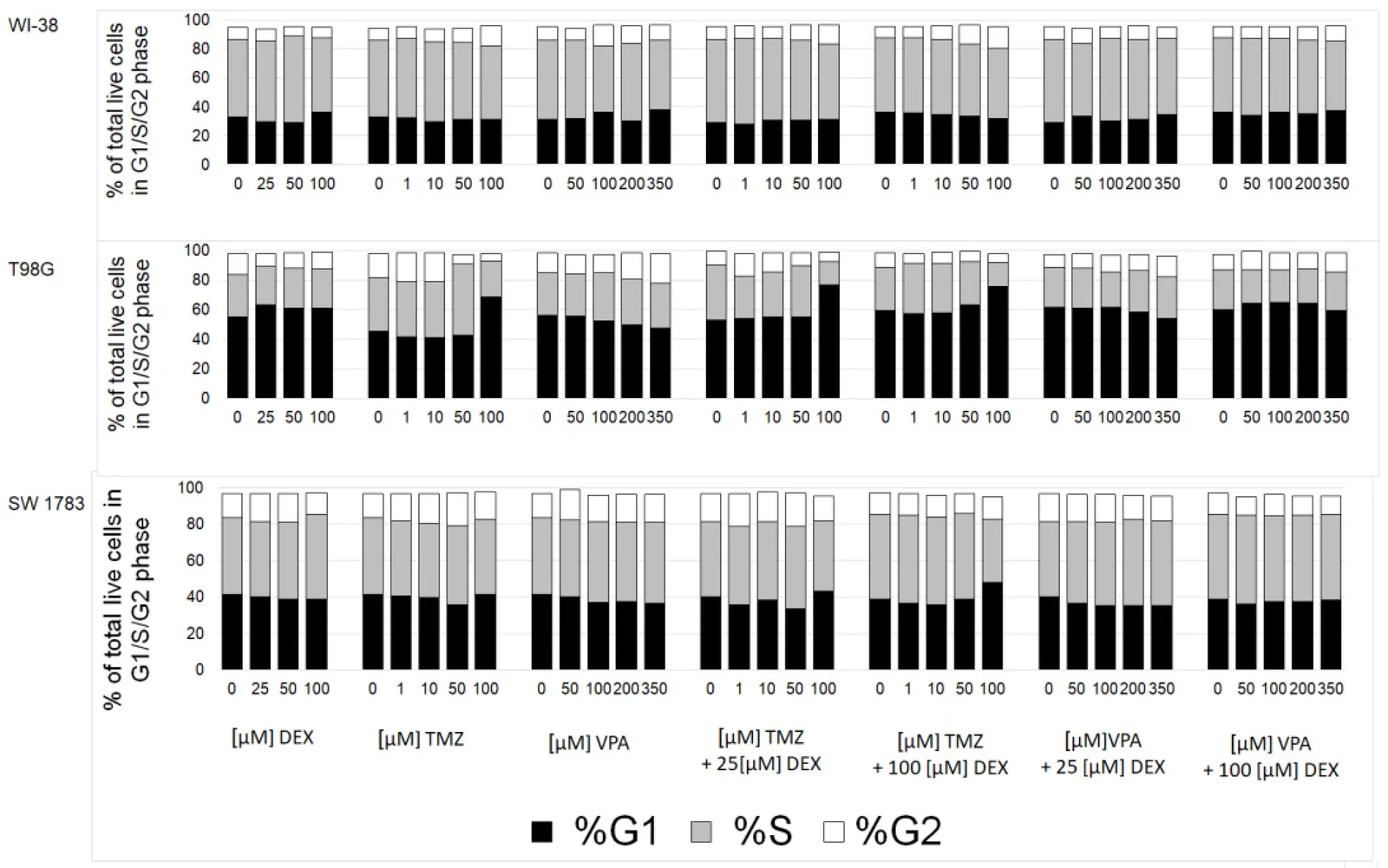
Effects of DEX, TMZ, and VPA on cell-cycle distribution in the WI-38, T98G, and SW1783 cells. Cells were treated for 24 h with the tested compounds alone or in selected combinations and analyzed by flow cytometry after propidium iodide staining. The figure shows the percentage of cells in the G1, S, and G2/M phases. Data were derived from a single experiment; no formal statistical comparison was performed.

**Figure 5:**
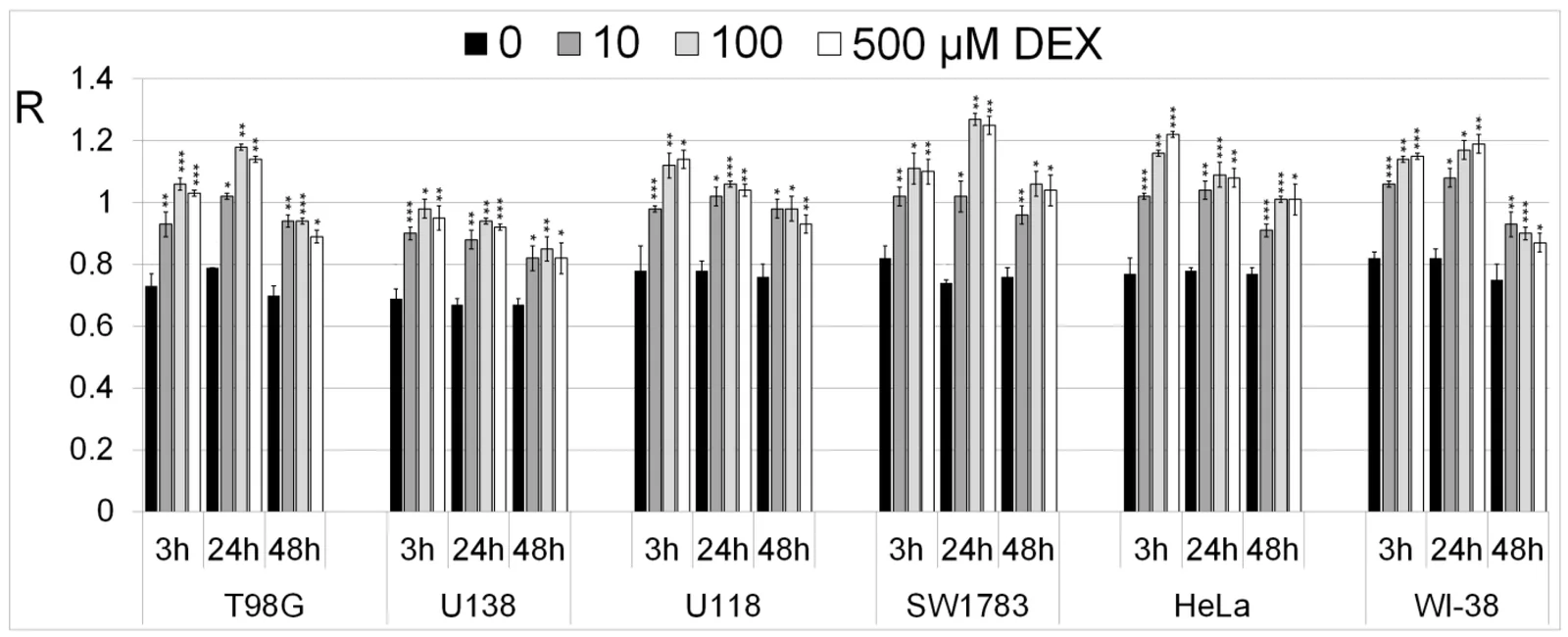
Effects of DEX on global DNA methylation measured as the total m^5^C content in glioma, HeLa, and WI-38 cell lines. Cells were treated with increasing concentrations of DEX, and m^5^C content was expressed as the R value. R was calculated as m^5^dC × 100/(m^5^dC + dC + dT). Deoxynucleotides were separated on cellulose-coated plates by two-dimensional thin-layer chromatography, and their amount was calculated using the ImageQuantTL 10.1 software. The analysis was performed after 3, 24, and 48 h of incubation at a given DEX concentration (0, 10, 100, 500 μM). For the control experiment, cells received H_2_O. Methylation levels differed significantly across DEX concentrations in all cell lines. The R values are the means from four independent experiments. Asterisks indicate a significant difference (* *p* < 0.05, ** *p* < 0.01, *** *p* < 0.001) from the control value.

**Figure 6:**
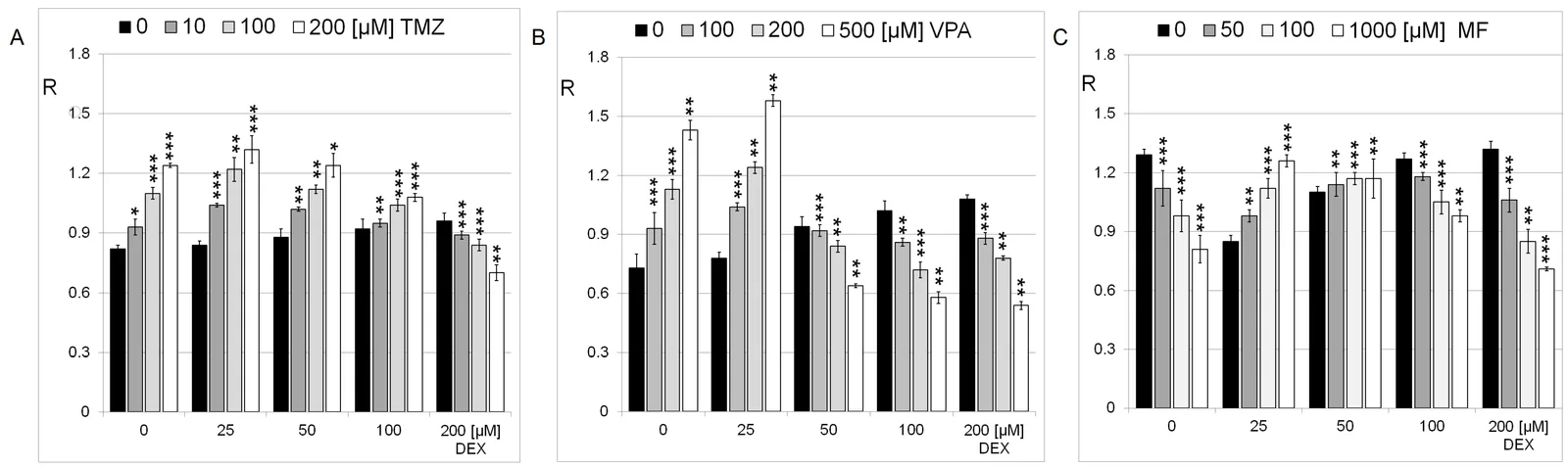
The effect of DEX in combination with TMZ, VPA and MF on the total DNA methylation level of the T98G cell line (**A**–**C**). The analysis was performed after 24 h of incubation at a given DEX concentration (25–200 µM), TMZ concentration (0–200 µM), VPA concentration (0–500 µM and MF concentration (0–1000 μM). The R values are the means from four independent experiments. Asterisks indicate a significant difference (* *p* < 0.05, ** *p* < 0.01, *** *p* < 0.001) from the control value.

**Figure 7:**
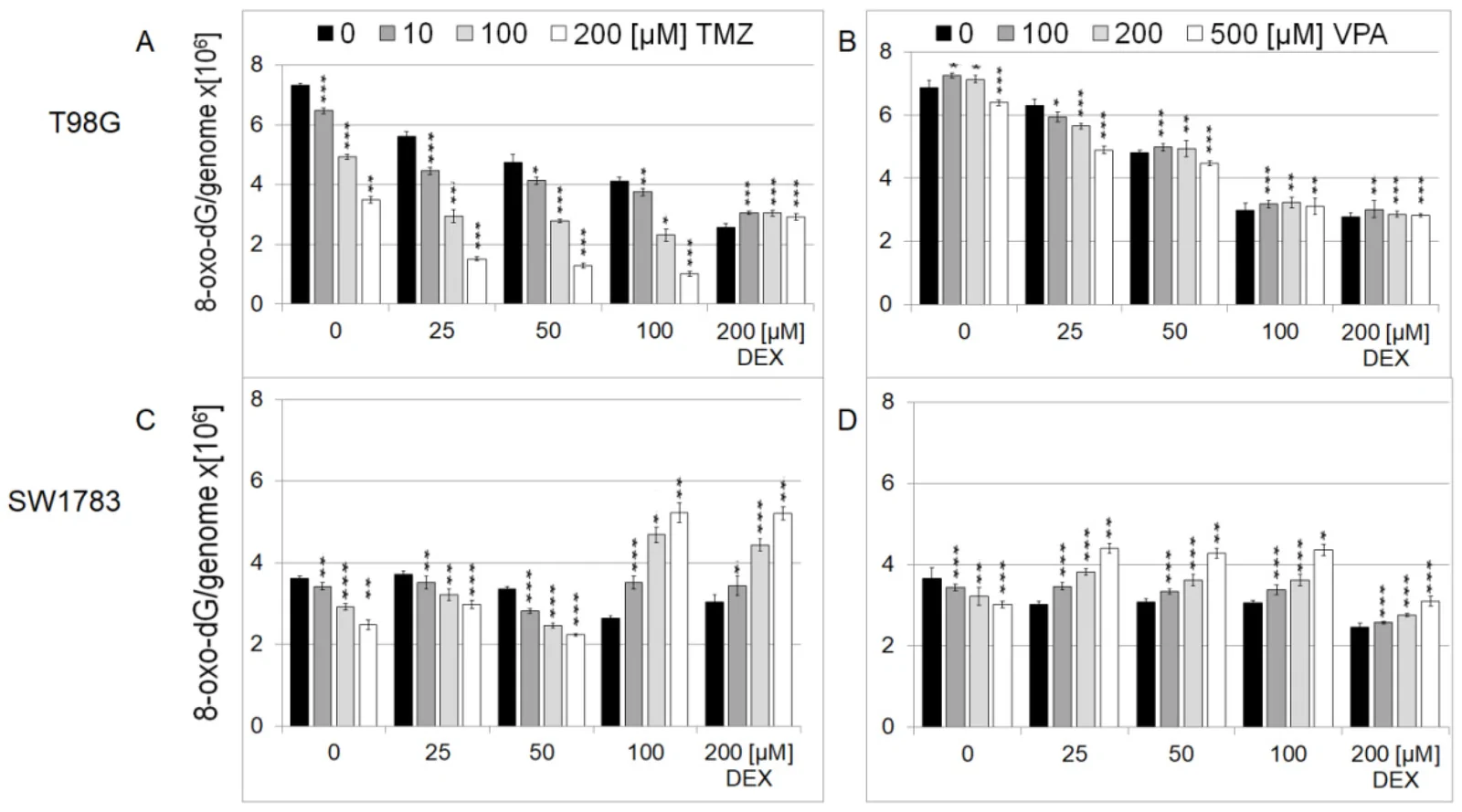
Effect of DEX in combination with TMZ and VPA on the 8-oxo-dG levels in T98G (**A**,**B**) and SW1783 (**C**,**D**) cell lines. Analysis was performed after 24 h of incubation. The 8-oxo-dG/genome values are the averages of four independent experiments. In the control experiment, cells were treated with H_2_O. Asterisks indicate a significant difference (* *p* < 0.05, ** *p* < 0.01, *** *p* < 0.001) from the control value.

**Figure 8:**
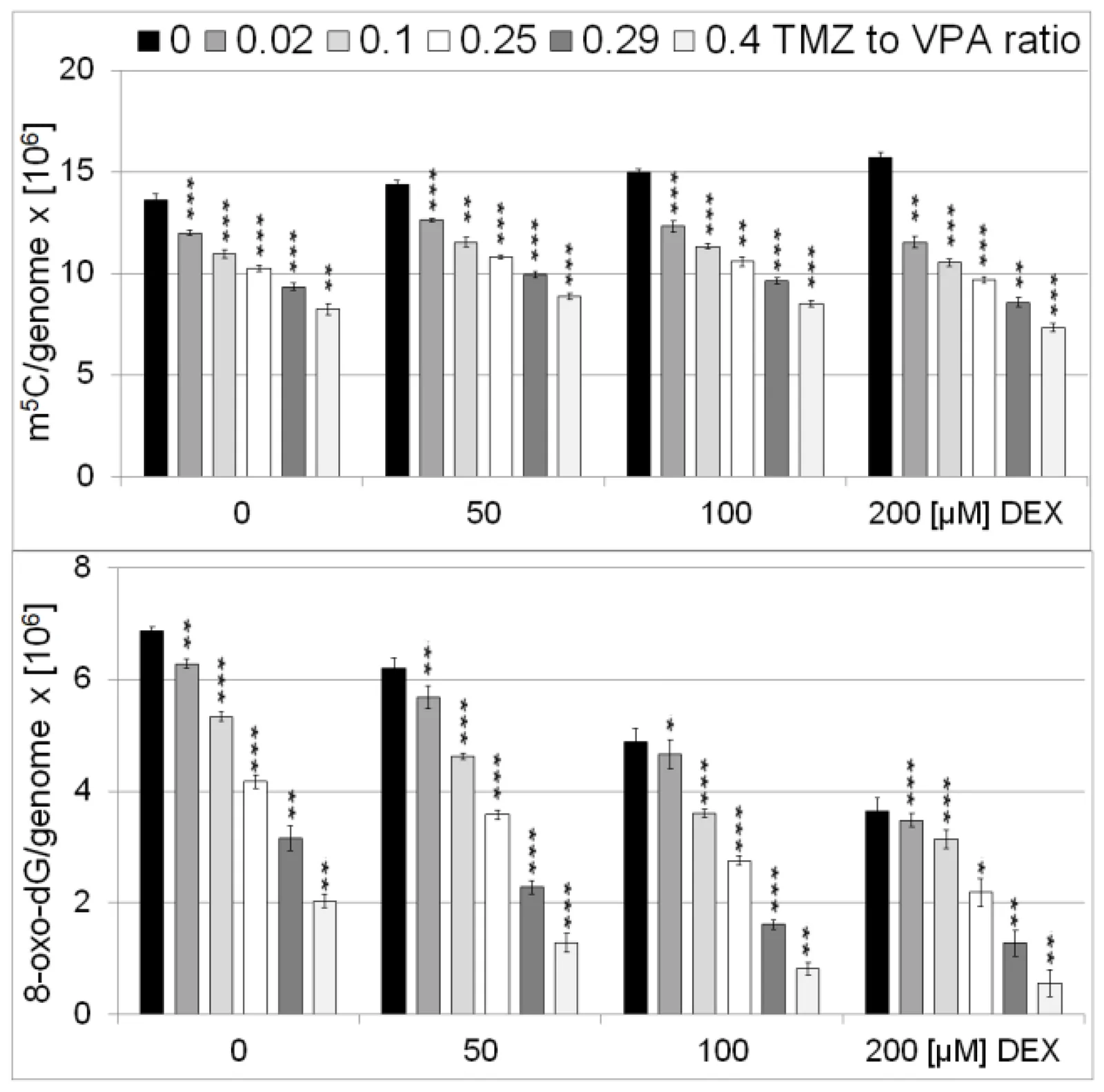
The effect of DEX in combination with a TMZ/VPA mixture at different ratios on the m^5^C and 8-oxo-dG contents in DNA for the T98G cell line. Incubation was for 24 h. DEX concentrations were 0, 50, 100, and 200 µM. The TMZ-to-VPA ratio ranged from 0 to 0.4. (0; 0.02—1/50; 0.1—10/100; 0.25—50/200; 0.29—100/350; 0.4—200/500 µM TMZ/µM VPA). The R values are the means from four independent experiments. Asterisks indicate a significant difference (* *p* < 0.05, ** *p* < 0.01, *** *p* < 0.001) from the control value.

## Data Availability

The original contributions presented in this study are included in the article. Further inquiries can be directed to the corresponding authors.

## Supplementary Materials

The following supporting information can be downloaded at: https://www.mdpi.com/article/10.3390/cells15191745/s1.
