# Supplementary material for: Dexamethasone Modulates Global DNA Methylation and Oxidative DNA Damage in Glioblastoma Cell Lines
Source: Cells. 2026 Sep 24;15(19):1745. doi: 10.3390/cells15191745 (PMC13664603; doi:10.3390/cells15191745)
Supplement: Supplementary file 1 [file cells-15-01745-s001.zip › Supplementary Table S1.pdf]

Supplementary Table S1. The statistical analysis of T98G glioblastoma cells after treatment with DEX, TMZ, VPA, and MF. Differences between the means of the test and control samples were assessed using 2-way ANOVA with multiple comparison, Tukey's multiple comparisons test. * $p<0.05$, ** $p<0.01$, *** $p<0.001$, **** $p<0.0001$

Fig. 1B (DEX, TMZ)

log conc of DEX	DEX		DEX, 1uM TMZ		DEX, 30uM TMZ		DEX, 100uM TMZ	
	Summary	Adjusted P Value	Summary	Adjusted P Value	Summary	Adjusted P Value	Summary	Adjusted P Value
0 vs 1.2	ns	0,6109	ns	0,9352	ns	0,9994	***	0,0004
0 vs 1.8	ns	>0,9999	ns	0,7987	ns	0,7286	**	0,0015
0 vs 2.4	****	<0,0001	****	<0,0001	****	<0,0001	****	<0,0001
0 vs 3.0	****	<0,0001	****	<0,0001	****	<0,0001	****	<0,0001

Fig. 1B (VPA, TMZ)

log conc of VPA	VPA		VPA, 1uM TMZ		VPA, 30uM TMZ		VPA, 100uM TMZ	
	Summary	Adjusted P Value	Summary	Adjusted P Value	Summary	Adjusted P Value	Summary	Adjusted P Value
0 vs 1.2	ns	0,7179	ns	0,0655	**	0,0011	ns	0,9997
0 vs 2.4	*	0,0229	****	<0,0001	ns	0,8894	ns	0,1168
0 vs 3.0	****	<0,0001	****	<0,0001	ns	0,5964	*	0,0346
0 vs 4.0	****	<0,0001	****	<0,0001	****	<0,0001	****	<0,0001

Fig. 1B (MF, TMZ)

log conc of MF	MF		MF, 1uM TMZ		MF, 30uM TMZ		MF, 100uM TMZ	
	Summary	Adjusted P Value	Summary	Adjusted P Value	Summary	Adjusted P Value	Summary	Adjusted P Value
0 vs 0.9	****	<0,0001	ns	0,2569	**	0,0038	ns	0,9971
0 vs 1.8	****	<0,0001	ns	0,1656	****	<0,0001	*	0,0164
0 vs 2.4	ns	0,3431	****	<0,0001	****	<0,0001	****	<0,0001
0 vs 3.3	***	0,0001	****	<0,0001	****	<0,0001	****	<0,0001

Fig. 1C

log conc of DEX	DEX		DEX, 50uM VPA		DEX, 350uM VPA		DEX, 500uM VPA	
	Summary	Adjusted P Value	Summary	Adjusted P Value	Summary	Adjusted P Value	Summary	Adjusted P Value
0 vs 0.6	ns	0,9501	ns	0,9246	****	<0,0001	***	0,0002
0 vs 1.2	ns	0,8466	ns	>0,9999	***	0,0004	****	<0,0001
0 vs 1.8	ns	>0,9999	ns	0,999	****	<0,0001	****	<0,0001
0 vs 2.4	****	<0,0001	***	0,0008	****	<0,0001	****	<0,0001
0 vs 3.0	****	<0,0001	****	<0,0001	****	<0,0001	****	<0,0001

Fig. 1D

log conc of DEX	DEX		DEX, 63uM MF		DEX, 500uM MF		DEX, 1000uM MF	
	Summary	Adjusted P Value	Summary	Adjusted P Value	Summary	Adjusted P Value	Summary	Adjusted P Value
0 vs 1.5	ns	0,5393	ns	0,9985	ns	0,723	ns	0,8955
0 vs 1.8	ns	0,7345	ns	0,9922	ns	0,7114	ns	0,9279
0 vs 2.0	*	0,0194	ns	0,5643	ns	0,2028	ns	0,188
0 vs 2.4	**	0,002	ns	0,4178	ns	0,9026	ns	0,9573
0 vs 3.0	*	0,0217	ns	0,0769	ns	0,2703	**	0,0045
