# Supplementary material for: Dexamethasone Modulates Global DNA Methylation and Oxidative DNA Damage in Glioblastoma Cell Lines
Source: Cells. 2026 Sep 24;15(19):1745. doi: 10.3390/cells15191745 (PMC13664603; doi:10.3390/cells15191745)
Supplement: Supplementary file 1 [file cells-15-01745-s001.zip › Supplementary Table S2.pdf]

Supplementary Table S2. The statistical analysis of real-time proliferation of T98G glioblastoma cells after treatment with DEX, TMZ, VPA, and their selected combinations. Differences between the means of the test and control samples were assessed using t-test, paired analysis. * p<0.05, ** p<0.01, *** p<0.001

Group 1	Control	Control	Control	Control	Control	Control	Control
vs	vs	vs	vs	vs	vs	vs	vs
Group 2	100 µM DEX	350 µM VPA	100 µM TMZ	100 µM DEX<br>100 µM TMZ	100 µM DEX<br>350 µM VPA	100 µM TMZ<br>350 µM VPA	100 µM DEX<br>100 µM TMZ<br>350 µM VPA
P value<br>(two-tailed P value)	0,0161	0,49	0,6199	0,0051	0,0066	0,8435	0,0461
P value summary	*	ns	ns	**	**	ns	*
Significantly different<br>(P < 0.05)?	Yes	No	No	Yes	Yes	No	Yes

Group 1	100 µM DEX	100 µM DEX	100 µM DEX	350 µM VPA	350 µM VPA	350 µM VPA	100 µM TMZ	100 µM TMZ	100 µM TMZ
vs	vs	vs	vs	vs	vs	vs	vs	vs	vs
Group 2	100 µM DEX<br>100 µM TMZ	100 µM DEX<br>350 µM VPA	100 µM DEX<br>100 µM TMZ<br>350 µM VPA	100 µM DEX<br>350 µM VPA	100 µM TMZ<br>350 µM VPA	100 µM DEX<br>100 µM TMZ<br>350 µM VPA	100 µM DEX<br>100 µM TMZ	100 µMTMZ<br>350 µM VPA	100 µM DEX<br>100 µM TMZ<br>350 µM VPA
P value<br>(two-tailed P value)	0,0004	0,0314	0,0005	0,0058	0,0049	0,0221	0,0041	0,002	0,0229
P value summary	***	*	***	**	**	*	**	**	*
Significantly different<br>(P < 0.05)?	Yes	Yes	Yes	Yes	Yes	Yes	Yes	Yes	Yes
